# Redefining interventional radiology: the urgent need for a clinical identity

**DOI:** 10.1186/s42155-026-00652-4

**Published:** 2026-02-03

**Authors:** Romaric Loffroy

**Affiliations:** Department of Vascular and Interventional Radiology, Image-Guided Therapy Center, François-Mitterrand University Hospital, Dijon, France

Interventional radiology (IR) has revolutionized patient care through minimally invasive, image-guided procedures. Yet, the specialty remains constrained by its historical association with diagnostic radiology. To ensure its survival and continued relevance, IR must evolve into a more clinically integrated discipline—one that assumes full responsibility for patient care, from consultation to follow-up. A paradigm shift toward interventional medicine is mandatory, encompassing changes in training, infrastructure, referral pathways, and nomenclature.

## From technical innovation to clinical identity

Since its emergence in the 1960s, IR has been synonymous with innovation. Image-guided angioplasty, embolization, and targeted tumor therapies have transformed medical and surgical practice [1]. However, while the field has advanced technologically, its professional identity has lagged behind. Many interventional radiologists remain embedded within diagnostic radiology departments, functioning primarily as procedural consultants rather than as longitudinal caregivers [2]. This structural dependency limits visibility, autonomy, and patient continuity. In contrast, proceduralists in other specialties—such as cardiology or gastroenterology—operate within clearly defined clinical frameworks. To thrive, IR must shed its image as a “service specialty” and redefine itself as a clinical specialty.

## Becoming true clinicians

The essential transformation involves moving from a procedural to a clinical model of care. Interventionalists must be doctors who treat, not merely operators who perform. That means establishing direct patient contact through

- Independent referral pathways: patients and referring physicians should have direct access to interventional specialists [3];
- Pre- and post-procedure consultations: building rapport and ensuring informed decision-making strengthen patient trust and satisfaction [4];
- Dedicated inpatient beds: allowing interventional teams to manage complications and optimize recovery [5];
- Structured follow-up programs: chronic diseases such as peripheral arterial disease or hepatocellular carcinoma require longitudinal care [6].

By embracing these principles, IR can align itself with the standards expected of any clinical specialty, improving outcomes and perception alike.

## The importance of a name

Language shapes perception. The term “interventional radiology” ties the specialty to its imaging roots, emphasizing tools over therapy. Renaming it “interventional medicine” would better reflect its purpose: using minimally invasive, image-guided procedures to diagnose and treat disease [7]. Like cardiology’s evolution from internal medicine or orthopedics from general surgery, interventional medicine could preserve collaboration with diagnostic radiology while asserting clinical independence. The change is more than cosmetic – “medicine” conveys accountability, care, and continuity. Patients understand it intuitively, and administrators recognize it as a service line rather than a support function.

## The limitations of the term “radiology”

“Radiology” no longer represents modern imaging’s breadth. Once limited to ionizing radiation, it now encompasses ultrasound, MRI, and hybrid modalities, many radiation-free. Retaining the term reinforces an outdated, narrow view that misrepresents today’s multimodal, clinically integrated imaging. It obscures how interventionalists use diverse technologies to guide therapy and manage disease. Reframing the field as interventional medicine would acknowledge this multimodality and its true mission: not to produce images, but to deliver care.

## Clinical integration: benefits across the system

Transforming IR into interventional medicine benefits all stakeholders. Patients gain continuity, direct communication, and clearer care pathways. Hospitals improve efficiency through streamlined admissions, dedicated beds, and reduced procedural bottlenecks. Referring physicians find a reliable clinical partner who takes ownership of outcomes rather than providing isolated technical assistance. Trainees experience a specialty with a defined identity and visible patient interaction, making recruitment more sustainable [8].

## Barriers to overcome

Several obstacles remain. Institutional structures, reimbursement models, and credentialing frameworks still bind IR to radiology. In many countries, board certification and training accreditation are controlled by radiologic societies. Shifting toward a clinical model will require collaboration, not confrontation, between interventional and diagnostic colleagues [9]. Cultural inertia is another challenge. Some radiologists fear that separation dilutes departmental cohesion. Yet, evolution does not equal isolation. Diagnostic radiology and interventional medicine can—and should—exist in symbiosis: one provides diagnosis, the other delivers therapy. Together, they complete the continuum of image-guided care.

## Training the next generation

Education must lead the transformation. Modern interventional training should encompass not only procedural skill but also clinical management, decision-making, and longitudinal follow-up [10]. Essential reforms include the following:

- Rotations in internal medicine, surgery, and critical care to strengthen clinical reasoning;
- Curriculum redesign emphasizing patient assessment and peri–procedural management;
- Mentorship and visibility—interventionalists should participate in ward rounds, tumor boards, and multidisciplinary clinics.

Medical students must see interventionalists as clinicians in white coats, not just operators behind lead glass. Exposure during training will help redefine IR in the minds of future doctors and patients alike.

## A call to action

As healthcare shifts toward patient-centered, value-based models, accountability and visibility are paramount. Specialties that remain invisible to patients risk marginalization. The path forward is clear: IR must claim its clinical identity, own its patients, and lead innovation not only in technology but also in care delivery. Rebranding as “interventional medicine” symbolizes this shift—a recognition that the specialty’s essence lies not in images, but in healing through precision and compassion. The transformation is already underway in leading centers worldwide [11]. Now is the time for our community to embrace it fully.

## Summary

This editorial calls for the transformation of interventional radiology into “interventional medicine” a fully clinical specialty that assumes complete responsibility for patient care. It argues that modern interventionalists must evolve beyond procedural roles, embracing longitudinal patient management, independent referral pathways, and clinical accountability. Such a shift, this author contends, will strengthen patient trust, professional identity, and the specialty’s long-term sustainability within a changing healthcare landscape.

## Data Availability

Not applicable.
